# Design of Liquid Crystal Materials Based on Palmitate, Oleate, and Linoleate Derivatives for Optoelectronic Applications

**DOI:** 10.3390/molecules28041744

**Published:** 2023-02-11

**Authors:** Salma A. Al-Zahrani, Mohd Taukeer Khan, Violeta Jevtovic’, Najat Masood, Yassin Aweis Jeilani, Hoda A. Ahmed

**Affiliations:** 1 Chemistry Department, Faculty of Science, University of Ha’il, P.O. Box 2440, Ha’il 81451, Saudi Arabia; 2 Department of Physics, Faculty of Science, Islamic University of Madinah, Al-Madinah Al-Munawwarah 42351, Saudi Arabia; 3 Department of Chemistry, Faculty of Science, Cairo University, Cairo 12613, Egypt; 4 Chemistry Department, Faculty of Science, Taibah University, Yanbu 46423, Saudi Arabia

**Keywords:** liquid crystals, optical properties, smectic stability, energy bandgap, fluorescence, binary phase diagram

## Abstract

Herein, liquid crystalline derivatives based on palmitate, oleate, and linoleate moieties with azomethine cores were synthesized, and their physical, chemical, optical, and photophysical properties were investigated in detail. The mesomorphic activity of these materials was examined through polarized optical microscopy (POM) and differential scanning calorimetry (DSC). The observed results revealed that the stability of the thermal mesophase depends on the terminal polar as well as on the fatty long-chain substituents. Purely smectogenic phases were detected in all three terminal side chains. A eutectic composition with a low melting temperature and a broad smectic A range was found by constructing a binary phase diagram and addressing it in terms of the mesomorphic temperature range. The energy bandgap of the palmitate-based derivative (**Ia**) was determined as 3.95 eV and slightly increased to 4.01 eV and 4.05 eV for the oleate (**Ib**) and linoleate (**Ic**) derivatives, respectively. The optical constants (n, κ, ε_r_, and ε_i_) were extracted from the fitting of measured spectroscopic ellipsometer data. The steady-state spectra of these samples exhibited a broad emission in the range 400–580 nm, which was found to be blue shifted to 462 nm for both **Ib** and **Ic** derivatives. The average fluorescence decay lifetime of the **Ia** derivative was found to be 598 ps, which became faster for the **Ib** and **Ic** derivatives and slower for the sample with a chloride end polar group.

## 1. Introduction

Optical liquid crystal (LC) materials are compounds that have a metaphase between solid and liquid phases. LC materials display one or more different mesophase according to the molecular ordering, such as layered smectic (Sm) and nematic (N) phases [1,2,3]. LCs can play an important role in a variety of applications, including displays, solar cells, sensors, modulators, optical imaging, etc., because of their properties [4,5]. Additionally suitable for use in biology, food production, medicine, and oil recovery are LCs. As a result, recent years have seen a rise in study into the development and design of liquid crystalline systems [6,7,8,9,10,11,12,13,14]. Three different forms of liquid crystalline materials can be distinguished: polymeric, lyotropic, and thermotropic. For their linear and nonlinear optical properties, thermotropic LCs have been extensively investigated and employed [14,15,16,17,18,19,20,21,22,23,24,25]. 

Numerous rod-like LC materials have been generated from Schiff bases and extensively examined [26,27,28,29,30,31]. Different impacts, including the orientation of the ester group within the rigid core, the position of the Schiff base unit with regard to the ester group, the lateral substitution [32], and the length of the terminal chain [33,34,35,36,37,38,39,40,41,42], have been studied. A terminal fatty acid substituent may stimulate and lower the melting temperature [43,44,45]. The resulting mesomorphic characteristics clearly illustrate this. In such a study, the polarity and direction of dipole moments change, but the molecular structure shows little change. As a result, a small modification to the shape of the molecule improves its optical properties, leading to novel mesophase behavior. When the length of the terminal substituent increases, the molecules tend to position themselves in a parallel configuration [46]. The length of the terminal chains also affects the twist-bend nematic and heliconical phases [47,48,49,50].

Typically, a multicomponent mixture with specific physical and chemical properties, namely, a wide temperature range of the mesophase, low viscosity, and the appropriate optical birefringence, is used to fill commercially available liquid crystal displays (LCDs). Only a few mesogenic and/or nonmesogenic chemical mixes may successfully satisfy these conditions. In order to design mixtures with the necessary properties, it is particularly necessary to understand the interactions between molecules of different components in liquid crystalline mixtures. Binary systems [23,42,45] have often been used to achieve extended liquid crystalline temperature ranges. In binary mixtures consisting of electron donors and acceptors, parallel molecular arrangement, required by liquid crystal formation, becomes observable if an electron donor–acceptor charge transfer interaction acts as the orientational force [51,52,53,54].

To find applications for liquid crystal materials in optoelectronic devices, it is vital to understand their optical and photophysical properties. In order to check whether the synthesized liquid crystalline materials are suitable for solar cells or optical sensors, it is important to evaluate the optical band gap, absorption coefficient, and lifetime of the charge carriers inside the material [55]. Moreover, to find applications for liquid crystalline materials in display devices, the knowledge of steady-state and time-resolved photoluminescence is requisite [56]. Furthermore, the optical parameters, including refractive index (n), extinction coefficient (κ), and real and imaginary parts of dielectric constants (ε_r_, ε_i_), are vital to determine the performance of the optoelectronic devices. The optical constant was evaluated by measurements with a spectroscopic ellipsometer.

Mesogenic materials, specifically as LCD displays, organic light-emitting diodes, anisotropic networks, photoconductors, and semiconductor materials, offer a wide range of valuable applications in science and technology [4]. Numerous mesogens, particularly thermotropic LCs, have been created as a result of the strong demand for new LCs for applications [28,29,30]. In earlier research, we found that the connecting units, azomethine and ester, were helpful for producing mesomorphism in thermotropic liquid crystals with two and three aromatic rings. Different polarity aromatic azomethine ester substitutes were shown to either enhance or reduce the mesomorphic characteristics [21]. 

In order to fully understand the correlation between the molecular structures of mesogens and their mesomorphic capabilities, several azomethine/ester homologous series of rod-like LCs have been described and frequently studied from the perspective of their alluring optical features [17]. These structural studies [26,27,28,29,30,31] can be used to determine the ester orientation inside a rigid core, the location of Schiff base/ester groups, lateral substitutions, and the terminal chain length. An LC compound’s molecules are strengthened by lateral substitution, which also contributes to the derivatives’ mesogenic properties. Through the disruption of molecular closed packing and a decrease in the LC melting point, the lateral substituent in the stiff core can successfully increase LC solubility.

Compounds that exhibit thermotropic mesomorphic properties widely vary in chemical constitution, but all of them possess a common feature: molecular geometric anisotropy. That is, the molecules of mesomorphic compounds are elongated and rod- or lath-like in shape, and this should be a necessary requirement. Compounds, such as normal paraffins and the homologues of acetic acid, are apparently geometrically suitable for mesophase formation but do not exhibit liquid crystalline properties. This is simply because the attractive forces operating between the molecules are insufficiently strong to maintain the parallel arrangement of the molecules after the crystal lattice has melted. In predicting whether a compound will be mesomorphic, the nature and probably strength of the intermolecular attractions must be considered, together with the purely geometric aspects of the molecule.

**Figure 1:**
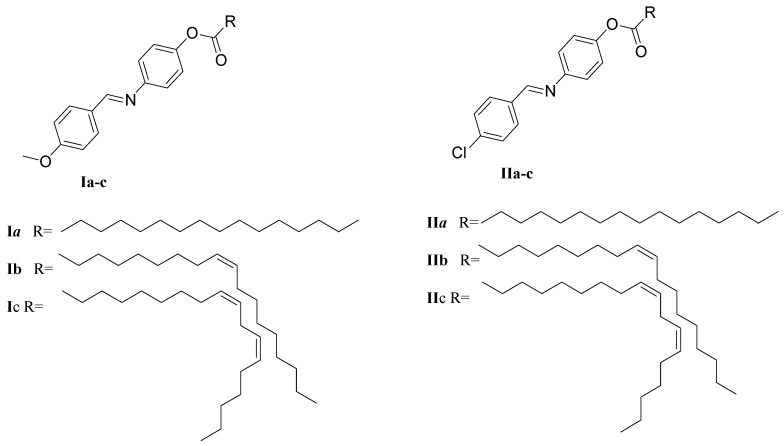
Schematic molecular structural of series **Ia-c** and **IIa-c**.

The goal of the present work was to design two different series of liquid crystalline derivatives based on palmitate, oleate, and linoleate moieties, and investigate them in detail. The two series differ from each other in the length of the terminal fatty chain attached to one end of the aromatic core, as shown in Figure 1. In the first series, a methoxy group (CH_3_O) was used [45], while in the second series, the methoxy group was replaced by chloro-substituent. The impact of the type of terminal group and the fatty chain length on their thermal, mesomorphic, and optical properties was investigated. The impact of the terminal fatty chain length and the terminal polar group (CH_3_O and Cl) on the optical and photophysical properties was investigated through the recording of the absorption spectra, spectroscopic ellipsometer measurements, and steady-state and time-resolved fluorescence spectra of the synthesized LC samples. 

## 2. Results and Discussion

### 2.1. Liquid Crystalline Behavior

**Figure 2:**
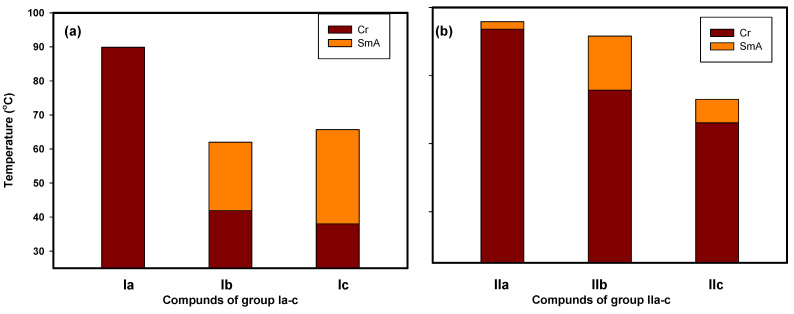
Transition temperature of mesophase behavior on heating of prepared derivatives for (**a**) **Ia-c** and (**b**) **IIa-c**.

**Figure 3:**
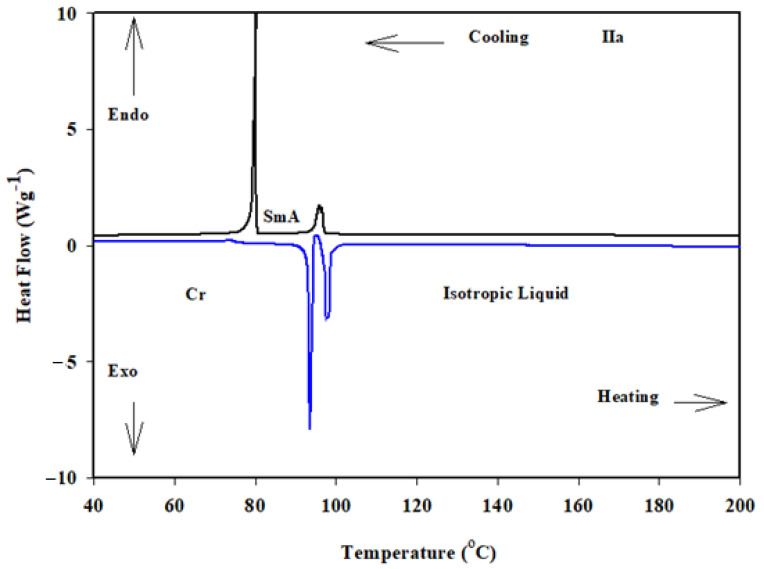
DSC thermograms of **IIa** derivative recorded from second heating and cooling at a rate of 10 ^o^C min^−1^.

**Figure 4:**
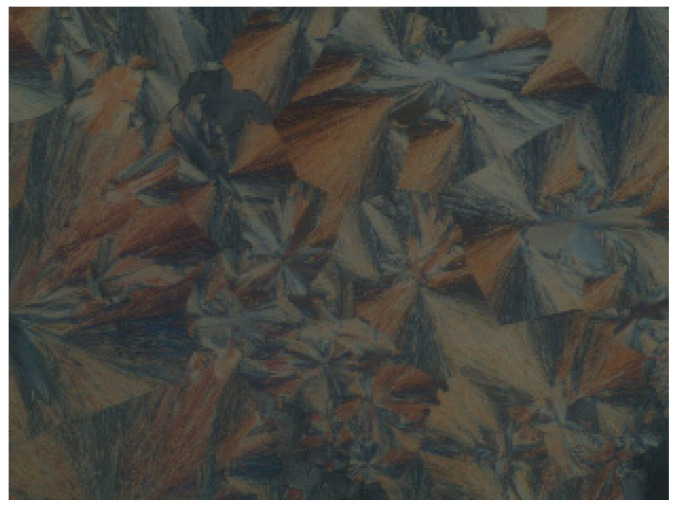
SmA texture of **IIa** derivative during cooling scan at 85 °C.

The transition temperatures and associated enthalpies of the synthesized materials were extracted from a DSC scan, which are tabulated in Table 1 and graphically depicted in Figure 2. The second heating scans were used to calculate the transition temperatures and enthalpy values. The stability of the heating and cooling DSC curves proved that all derivatives were thermally stable. Figure 3 illustrates the DSC heating and cooling traces of compound **IIa** as an example. The POM measurements showed textures that supported the formation of mesophases (Figure 4).

As can be seen in Table 1, all compounds from the **Ia-c** and **IIa-c** series displayed enantiotropic LC phases, except for the **Ia** derivative, which showed a monotropic phase. Moreover, all of them exhibited thermal mesophases in different temperature ranges depending on the nature of the terminal fatty chain. To determine the effect of the length of the terminal fatty chain as well as the terminal polar group (CH_3_O and Cl) on the mesophase behavior in each series, the transition temperatures of all the examined derivatives are graphically displayed in Figure 2.

For the polar CH_3_O group (**Ia-c**) [45] that donated electrons (Table 1 and Figure 2), all of the produced compounds proved the presence of the smectic A mesophase. Compound **Ia** had a limited monotropic smectic A phase range of 9.8 °C and was completely smectogenic. A wider SmA temperature range than that of the saturated counterpart **Ia** (20.1 °C) was observed because of the length and unsaturation of the wing group in compound **Ib** [45]. In general, mesophase stability was enhanced by increasing the polarizability and/or polarity of the entire molecule’s mesogenic component. Compound **Ic** expanded the SmA temperature range (to 27.7 °C) by means of its longer terminal chain compared with that of compound **Ia** and two conjugated double bonds above its corresponding analogue **Ib**.

Changing the terminal polar group X from the methoxy to the chloro led to the formation of the second series of materials **IIa-c**. As can be seen from Table 1 and Figure 2, the melting temperatures were reduced for all derivatives because of the dilution effect caused by the longer fatty chain. Essentially, the same phase type observed in **Ia-c** was also exhibited by **IIa-c**. Additionally, all of the members of the homologous series **IIa-c** were enantiotropic with mesophase thermal stability greater than that of group **Ia-c**. The SmA range decreased when replacing the X from methoxy to the Cl moiety. Figure 1 also illustrates that the presence of one or two double bonds resulted in a reduction in the smectic A stabilities and melting temperatures. The single double bond in structure **IIb** or the two conjugated double bonds in structure **IIc**, which were separated from the core mesogenic group, inhibited conjugation with the central linking azomethine group. The main potential cause of this decline in smectic A phase stability is a nonlinear cis-trans effect [43,44,45].

### 2.2. Binary Mixtures

**Figure 5:**
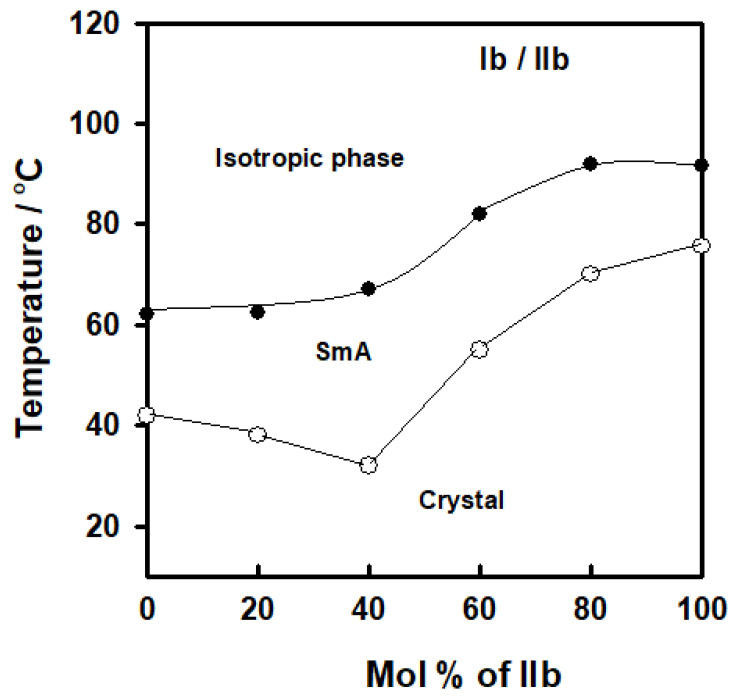
Binary phase diagram of **Ib/IIb** system.

A sample binary phase diagram for two components with identical fatty chain lengths and differing terminal polar groups X is shown in Figure 5. The binary mixtures were produced from the enantiotropic terminal chain homologues (**Ib/IIb**). Both derivatives showed an enantiotropic SmA mesophase. In this figure, the binary phase diagram shows a slight increase in the SmA phase relative to the expected behavior. The differences in polarity between the two components of the combination that were attracted to one another and the improved the arrangement of the molecules were responsible for the minor special properties of the smectic A phase. Figure 5 also demonstrates that a solid mixture with a eutectic composition of 40.0 mol % of **IIb** had a eutectic melting point of 31.8 °C and a mesomorphic temperature range of 35.1 °C. It may be deduced that the incorporation of the terminal polar groups (CH_3_O and Cl) influenced both the conformation and steric effect in pure and mixed states and led to the formation of a wide SmA phase. 

### 2.3. Optical Properties

**Figure 6:**
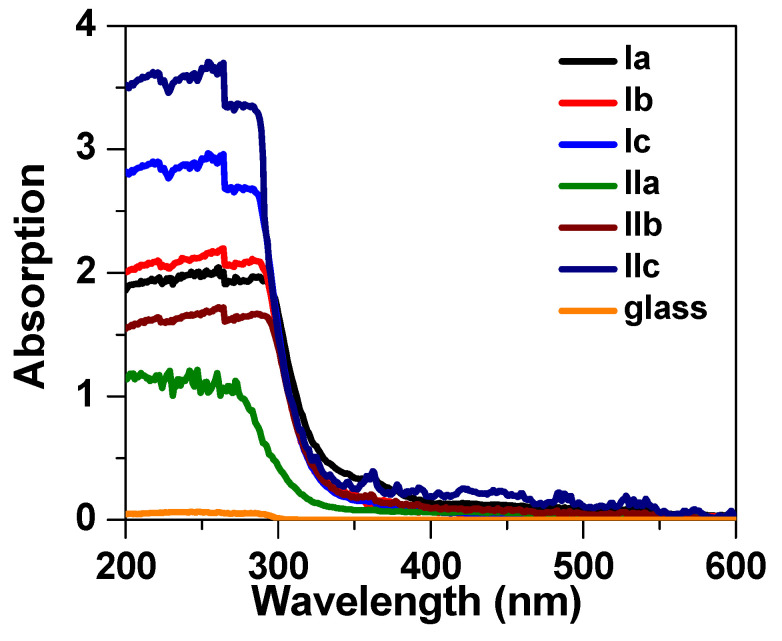
Absorption spectra of the liquid crystalline solid films on glass slides.

**Figure 7:**
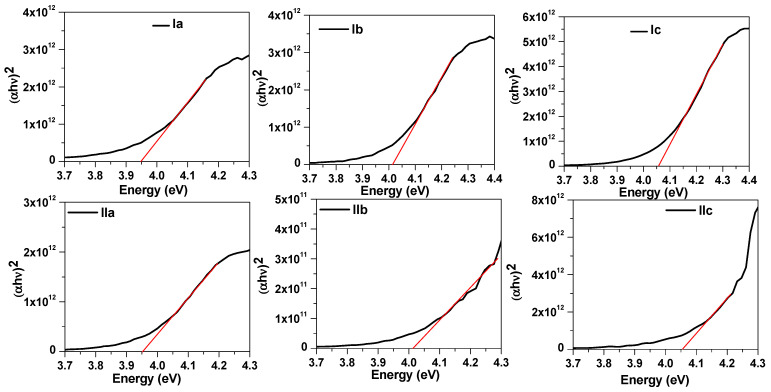
Tauc plots of synthesized liquid crystalline materials.

The optical properties of the liquid crystalline materials were recorded by preparing thick films on glass slides. The absorption spectra were recorded with an Agilent Cary 5000 UV–Vis–NIR spectrophotometer. The absorption spectra of these films are shown in Figure 6. It can be noticed that these films exhibited a broad absorption in the UV region corresponding to the π → π* transition in the aromatic ring [57,58]. All liquid materials displayed similar absorbance spectra, and absorbance became zero for wavelengths greater than 350 nm. The energy bandgap (*E_g_*) of the synthesized liquid crystalline materials was evaluated through Tauc’s relation [59]: (*αhv*) = *B*(*E* − *E_g_*), where *α* is the absorption coefficient, *B* is an empirical constant, and *E* is the energy of the incident light. The absorption coefficient *α* was calculated by the relation [60]: $\alpha =\frac{\ln(10)A}{L}$, where *A* and *L* are the absorbance and thickness of the sample, respectively. The energy bandgap was extracted by the intercept of the tangent to the energy axis, as illustrated in Figure 7, with corresponding values listed in Table 2. The energy bandgap of (4-methoxybenzylideneamino) phenyl palmitate was determined to be 3.95 eV, which slightly increased to 4.01 eV and 4.05 eV for the (4-methoxybenzylideneamino) phenyl oleate (**Ib**) and (4-methoxybenzylideneamino) phenyl linoleate (**Ic**) samples, respectively. The increase in *E_g_* values was due to the bending of the alkyl side chain [61] in these two samples, whereas the fatty side chain was straight in the first sample, as illustrated in the chemical structure shown in Figure 1. However, no significant change in the absorption spectra was noticed when replacing the methoxy group with chloride; even the energy band gap was found to be similar, indicating end groups had no significant impact on the absorbance spectra. 

**Figure 8:**
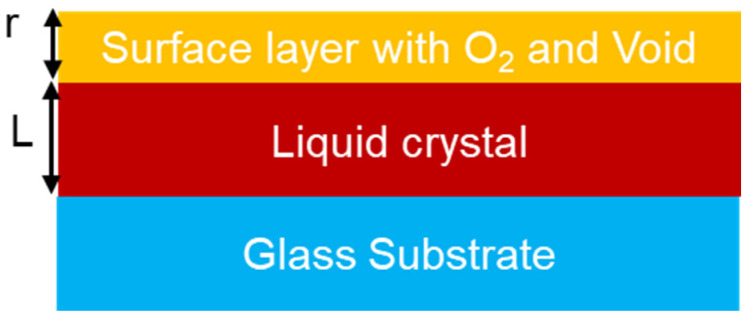
The optical model used to extract the optical constants of liquid crystalline thin films.

**Figure 9:**
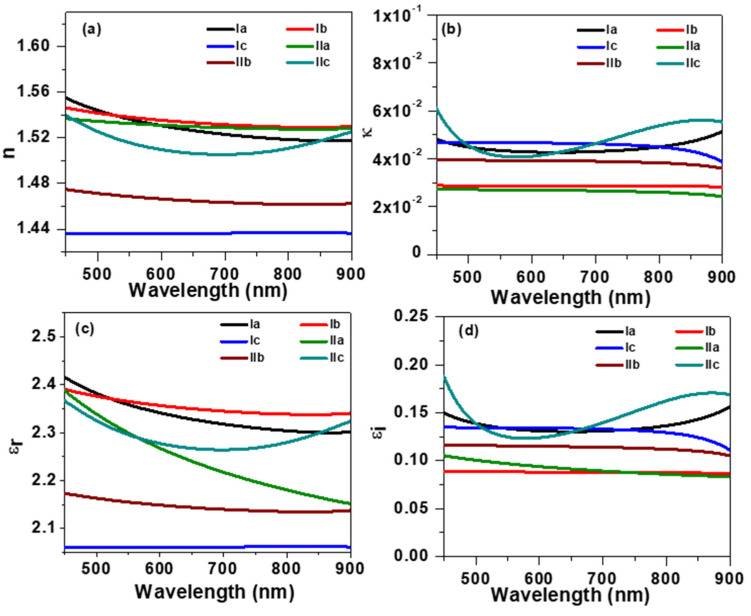
The optical constants of liquid crystalline materials extracted with spectroscopic ellipsometer measurement; (**a**) refractive index; (**b**) extinction coefficient; (**c**,**d**) dielectric contents.

The spectroscopic ellipsometer is a tool used to indirectly determine the optical parameters of a thin film. It measures the change in the amplitude ratio (*ψ*) and phase difference (Δ) of the reflected elliptical polarized light through the sample. The Fresnel reflection coefficient *ρ* is defined through the equation [62,63,64,65]: *ρ* = tan*ψe*^*i*Δ^. The measured spectroscopic data were analyzed through the optical model shown in Figure 8, and the extracted optical parameters as a function of wavelength are shown in Figure 9. 

The complex refractive index is defined as $\tilde{N}=n-i\kappa$, where *n* is the refractive index, defined by the ratio of the speed of light in a vacuum to the speed of light inside the material; *κ* is the extinction coefficient, illustrating the loss of optical energy inside the materials. It can be noticed from Figure 9a that the refractive index of (4-methoxybenzylideneamino) phenyl palmitate was 1.56 at 450 nm, which slightly decreased with the increase in wavelength. The refractive index of (4-methoxybenzylideneamino) phenyl oleate samples was found to be more stable than that of **Ia**; however, it dropped below 1.44 for the (4-methoxybenzylideneamino) phenyl linoleate liquid crystalline sample. The refractive index of the second series was found to be lower than that of the first series. The extinction coefficient of the liquid crystalline material shown in Figure 9b was very low in the 450–900 nm region because these materials do not absorb much light in this region, as shown in Figure 6. The real part of dielectric constant ε_r_ is shown in Figure 9c. The ε_r_ values of samples **Ia** and **Ib** were found to be 2.42 and 2.39 at 450 nm, respectively, which slightly decreased for higher wavelengths. However, the value of ε_r_ was very low for sample **Ic** and dropped to 2.06 and remained constant at higher wavelengths. The dielectric constant of **IIa** sample, i.e., replacing terminal polar groups from CH_3_O to Cl, was found to be 2.39 at 450 nm and decreased faster than that of the same sample with the polar group CH_3_O. Samples **IIb** and **IIc** had lower a dielectric constant than **IIa**.

### 2.4. Photophysical Properties

**Figure 10:**
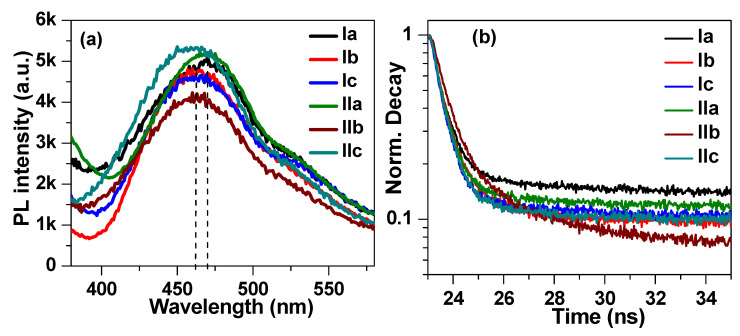
(**a**) Steady-state photoluminescence and (**b**) time-resolved fluorescence spectra of liquid crystalline material.

To understand the photophysical phenomenon in liquid crystalline materials, the steady-state and time-resolved fluorescence (TRF) spectra were recorded with a DeltaFlex time correlated single photon counting (TCSPC) system from Horiba (Horiba Instruments, Piscataway, United States). The steady-state spectra were recorded by exciting the sample with a delta-diode wavelength λ = 319 ± 10 nm, whereas the decay was monitored at 470 nm. The steady-state spectra shown in Figure 10a exhibited a broad emission in the range of 400 nm–580 nm associated with the π^∗^→π emission of the benzene ring. The peak emission for (4-methoxybenzylideneamino) phenyl palmitate sample (**Ia**) was observed at 470 nm, which blue-shifted to 462 nm for both the (4-methoxybenzylideneamino) phenyl oleate (**Ib**) and (4-methoxybenzylideneamino) phenyl linoleate (**Ic**) samples, shown by dotted lines. The blue shift in the emission spectra was associated with the bending of a long side chain in phenyl oleate (**Ib**) and phenyl linoleate (**Ic**) samples. It can be noticed from Figure 10a that no significant change in emission spectra was observed by replacing the methoxy group with a chloride group. The TRF spectra of the synthesized liquid crystalline materials are shown in Figure 10b, which were recorded at 470 nm. The spectra were fitted with a biexponential decay function: *I*(*t*) = *A* + *B*_1_*e*^−*t*/*τ*_1_^ + *B*_2_*e*^−*t*/*τ*_1_^, where *τ*_1_ and *τ*_2_ are the lifetime attributed to different photophysical phenomena; *A* is an empirical constant; and *B*_1_ and *B*_2_ represent the proportion of electrons with lifetime of *τ*_1_ and *τ*_2_, respectively. The fitting parameters are listed in Table 2. It can be noticed that for all samples, the lifetime was in the ps range, and both *τ*_1_ and *τ*_2_ had similar values, indicating that they had the same origin. The average lifetime was calculated through the relation [62,63]: $\tau_{\mathrm{avg}}=\frac{B_{1}\tau_{1}+B_{2}\tau_{2}}{B_{1}+B_{2}}$. The average lifetime of the **Ia** sample was evaluated to be 598 ps. The decay was slightly faster for **Ib** and **Ic,** with lifetimes of 548 ps and 518 ps, respectively. Furthermore, the series with chloride end polar groups had a slightly longer lifetime. The liquid crystalline sample (4-chlorobenzylideneamino) phenyl palmitate (**IIa**) had the highest lifetime among the six samples, with an average lifetime of 892 ps. The lifetime of samples **IIb** and **IIc** had lifetimes similar to those of **Ib** and **Ic,** with an average lifetime of 532 ps and 523 ps, respectively. 

## 3. Experimental Section

All materials used are given in Supplementary Materials.

### 3.1. Synthesis of Materials

**Scheme 1:**
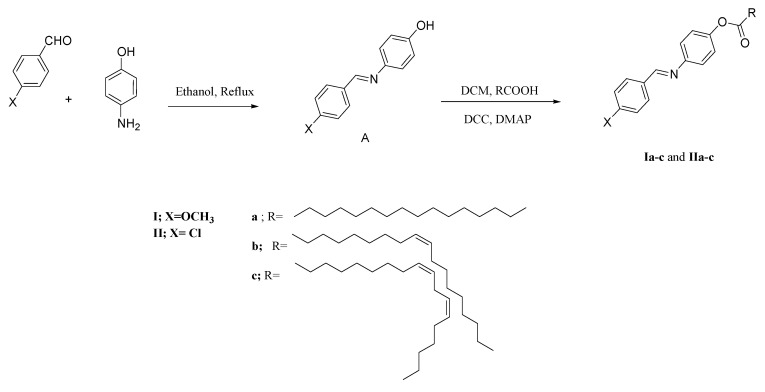
Synthesis of groups **Ia-c** and **IIa-c**.

Series **Ia-c** and **IIa-c** were formed according to the following Scheme 1.

#### 3.1.1. Synthesis of (4-Substituted Benzylideneamino) phenol (A)

In 10 mL of ethanol, 4.1 mmol of each of (4-substitutedbenzaldehyde) and (4-aminophenol) were refluxed for two hours. The separated product was filtered when the reaction mixture had cooled. From ethanol, the resulting solid was recrystallized.

#### 3.1.2. Synthesis of Fatty acid Derivatives, **Ia-c** and **IIa-c**

In 25 mL of dry methylene chloride, equimolar quantities of 4-substituted benzylideneamino phenol (A, 4.1 mmol) and 4-fatty acids (4.1 mmol each) were dissolved. A very little amount of crystals of 4-dimethylaminopyridine (DMAP) served as a catalyst, and N,N′-dicyclohexylcarbodiimide (DCC, 0.02 mole) was added. The mixture was continuously stirred while being allowed to stand for 72 hours at room temperature. To produce TLC pure products, the obtained solid residue was filtered out and recrystallized twice from ethanol. TLC’s TLC chromatogram showed a single distinct spot, and its differential scanning calorimetry (DSC) thermograms showed sharp melting and clearing peaks. This approach has already been described [45].

### 3.2. Characterization

Using TLC sheets from Merck (Sigma-Aldrich Chemie GmbH, Taufkirchen Germany) coated in silica gel, spots were detected by UV irradiation.

Tetramethyl silane was employed as an internal standard in a 500 MHz Varian EM 350 L spectrometer (Oxford, UK), and the chemical shift values were recorded in parts per million. Elemental studies were performed using a Thermo Scientific Flash 2000 CHS/O Elemental Analyzer (Milan, Italy).

Phase transitions were recorded using a TA Instruments Co. Q20 Differential Scanning Calorimeter (DSC) (New Castle, DE 19720, USA) device. Lead and indium were used to calibrate the melting temperatures and enthalpies for DSC. For the DSC investigation, samples weighing 2–4 mg were employed in aluminum pans. The rate of heating was 10 °C/min in an inert atmosphere of nitrogen gas (30 mL/min). All transition temperatures were recorded during the second heating scan, which involved heating the atmosphere to 300 °C and then cooling it to 0 °C. 

A polarized optical microscope (POM, Wild, Germany) attached to a Mettler FP82HT hot stage was used to check the transition temperatures for the produced compounds and to identify phases.

Using an Agilent Cary 5000 UV–Vis–NIR spectrophotometer, the absorption spectra of the produced films were recorded. By excitation, the sample from a Delta-diode at 320 nm with a peak width of 10 nm and a Horiba delta flex TCSPC lifetime fluorometer was used to record the steady-state emission and time-resolved decay spectra. A Horiba Smart-SE spectroscopic ellipsometer was employed to evaluate the refractive index (n), extinction coefficient (k), and dielectric constants of liquid crystalline samples.

Examples of characterizations

**Ia:** Yield = 92.0%, m.p. = 90.0 °C, FTIR (υ’, cm^−1^): 2924–2865 (CH2 stretching), 1739 (C=O), 1613 (C=N), 1604 (C=C), 1472 (C−OAsym), 1232 (C–OSym). ^1^H NMR (500 MHz, DMSO-d_6_) δ 8.63 (s, 1H, CH=N), 7.93 (d, 2H, Ar-H), 7.57 (d, 2H, Ar-H), 7.30 (d, 2H, Ar-H), 7.14 (d, 2H, Ar-H), 3.90–3.10 (m, 3H), 2.50 (m, 2H, CH2), 1.68–1.59 (m, 2H, CH2), 1.34–1.22 (m, 24H, 12CH2), 0.82 (t, 3H, CH3). Anal. Calc.: C, 77.38; H, 9.31; N, 3.01. Found: C, 77.35; H, 9.30; N, 3.00.

**Ib:** Yield = 94.7%, m.p. = 41.9 °C, ^1^H NMR (600 MHz, DMSO) δ 8.37 (s, 1H), 7.85 (d, *J* = 8.7 Hz, 2H), 7.20 (d, *J* = 8.6 Hz, 2H), 7.09 (d, *J* = 8.6 Hz, 2H), 6.99 (d, *J* = 8.7 Hz, 2H), 5.98 (m, 1H), 5.36 (m, 1H), 3.87 (s, 3H), 2.55 (t, *J* = 7.5 Hz, 2H), 2.11–1.95 (m, 4H), 1.78–1.69 (m, 2H), 1.53–1.07 (m, 22H), 0.87 (t, *J* = 6.9 Hz, 3H). ^13^C NMR (151 MHz, DMSO) δ = 169.24, 158.86, 156.44, 146.42, 145.04, 127.24, 126.76, 126.46, 125.70, 118.93, 118.35, 110.91, 52.25, 30.99, 30.56, 28.55, 26.42, 26.34, 26.18, 25.98, 25.81, 25.75, 25.72, 23.84, 22.27, 21.62, 19.38 Anal. Calc.: C, 78.17; H, 9.22; N, 2.85. Found: C, 78.16; H, 9.20; N, 2.84.

## 4. Conclusions

Two liquid crystal series that differed from each other in the length of the terminal fatty chain and terminal polar compact substituent were prepared and thermally investigated. The experimental mesomorphic and optical properties for all prepared materials and their binary mixtures revealed that the terminal polar groups played an important role in the thermal stabilities of the prepared compounds. All materials showed a purely SmA phase. Moreover, a low melting temperature with a broad smectic A range was detected at the eutectic composition of their binary phase diagram. The energy bandgap was found to slightly increase with the increase in the fatty side chain length; however, no significant change in bandgap was observed when replacing the terminal polar groups from CH_3_O to Cl. The optical constants of two series were extracted from the fitting of spectroscopic ellipsometer data, which were found to be lower for the samples with a longer fatty chain as well as for Cl end polar group samples. The synthesized liquid crystalline materials exhibited a broad PL emission with charge carrier lifetime of the order of ps, which was found to decay faster for the longer fatty side chain derivatives. The obtained results revealed that the synthesized materials can be employed in display devices, but further investigation is required to check how the optical property varies with electrical signal. 

## Figures and Tables

**Table 1:** Transition temperature (°C), enthalpy (kJ/mol), normalized entropy of transition, and mesophase range of groups **Ia-c** and **IIa-c**.

Compound	*X*	*T*_Cr_-SmA	∆*H*_Cr_-SmA	*T*_SmA_-I	∆*H*_SmA_-I	∆*T*_SmA_	∆*S/R*
**Ia**	CH_3_O	89.9	32.93	88.8 *	2.69	9.8 *	0.89
**Ib**	CH_3_O	41.9	35.78	62.0	2.98	20.1	1.07
**Ic**	CH_3_O	38.0	37.23	65.7	2.20	27.7	0.78
**IIa**	Cl	93.6	40.37	95.8	2.34	2.2	0.76
**IIb**	Cl	75.7	38.03	91.6	2.71	15.9	0.89
**IIc**	Cl	66.1	32.33	73.0	1.65	6.9	0.57

Abbreviations: Cr-SmA = transition from solid to the SmA phase; SmA-I = transition from SmA to the isotropic phase. * monotropic SmA phase.

**Table 2:** Optical bandgap and fitting parameters of decay spectra for series **Ia-c** and **IIa-c**.

Films	*E_g_* (eV)	*A*	*τ*_1_ (ps)	*B*_1_	*τ*_2_ (ps)	*B*_2_	*τ*_avg_ (ps)	χ^2^
**Ia**	3.95	734	630	0.81	465	0.19	598	1.84
**Ib**	4.01	1288	560	0.73	514	0.27	548	1.20
**Ic**	4.06	946	518	0.79	517	0.21	518	1.72
**IIa**	3.95	751	996	0.82	420	0.18	892	1.21
**IIb**	4.02	913	535	0.80	519	0.20	532	1.18
**IIc**	4.05	979	526	0.77	512	0.23	523	1.67

## Data Availability

The data presented in this study are available on request from the corresponding author.

## Supplementary Materials

The following supporting information can be downloaded at: https://www.mdpi.com/article/10.3390/molecules28041744/s1, the material details of the investigated compounds.

## References

[1] De Gennes P.-G., Prost J. (1993). The Physics of Liquid Crystals.

[2] Chandrasekhar S. (1994). Liquid Crystals.

[3] Priestly E. (2012). Introduction to Liquid Crystals.

[4] Kraft A., Reichert A., Kleppinger R. (2000). Supramolecular liquid crystals with columnar mesophases through self-assembly of carboxylic acids around a tribasic core. Chem. Commun..

[5] Fleischmann E.K., Zentel R. (2013). Liquid-crystalline ordering as a concept in materials science: From semiconductors to stimuli-responsive devices. Angew. Chem. Int. Ed..

[6] Laschat S., Baro A., Steinke N., Giesselmann F., Haegele C., Scalia G., Judele R., Kapatsina E., Sauer S., Schreivogel A. (2007). Discotic liquid crystals: From tailor-made synthesis to plastic electronics. Angew. Chem. Int. Ed..

[7] Tschierske C. (2013). Development of structural complexity by liquid-crystal self-assembly. Angew. Chem. Int. Ed..

[8] Rosu C., Maximean D.M., Kundu S., Almeida P.L., Danila O. (2011). Perspectives on the electrically induced properties of electrospun cellulose/liquid crystal devices. J. Electrost..

[9] Gilli J., Thiberge S., Manaila-Maximean D. (2004). New aspect of the voltage/confinement ratio phase diagram for a confined homeotropic cholesteric. Mol. Cryst. Liq. Cryst..

[10] Ibrahim M.F., Senior S.A., El-atawy M.A., El-Sadany S.K., Hamed E.A. (2011). Dft calculations of 2,4,6-trinitrophenylbenzoate derivatives: Structure, ground state properties and spectral properties. J. Mol. Struct..

[11] Chen K.-y. (2015). Crystal Structure, Hydrogen-Bonding Properties, and DFT Studies of 2-((2-(2-Hydroxyphenyl) benzo [d] thiazol-6-yl) methylene) malononitrile. Mol. Cryst. Liq. Cryst..

[12] Shoji M., Tanaka F. (2002). Theoretical study of hydrogen-bonded supramolecular liquid crystals. Macromolecules.

[13] Sundaram S., Jayaprakasam R., Dhandapani M., Senthil T., Vijayakumar V. (2017). Theoretical (DFT) and experimental studies on multiple hydrogen bonded liquid crystals comprising between aliphatic and aromatic acids. J. Mol. Liq..

[14] Nafee S.S., Hagar M., Ahmed H.A., Alhaddad O., El-Shishtawy R.M., Raffah B.M. (2020). New two rings Schiff base liquid crystals; ball mill synthesis, mesomorphic, Hammett and DFT studies. J. Mol. Liq..

[15] El-Atawy M., Naoum M., Al-Zahrani S., Ahmed H. (2021). New Nitro-Laterally Substituted Azomethine Derivatives; Synthesis, Mesomorphic and Computational Characterizations. Molecules..

[16] Ahmed N.H., Saad G.R., Ahmed H.A., Hagar M. (2020). New wide-stability four-ring azo/ester/Schiff base liquid crystals: Synthesis, mesomorphic, photophysical, and DFT approaches. RSC Adv..

[17] Alhaddad O.A., Khushaim M.S., Gomha S.M., Ahmed H.A., Naoum M.M. (2022). Mesophase behavior of four ring ester/azomethine/ester liquid crystals in pure and mixed states. Liq. Cryst..

[18] Nafee S.S., Ahmed H.A., Hagar M. (2020). New architectures of supramolecular H-bonded liquid crystal complexes based on dipyridine derivatives. Liq. Cryst..

[19] Ahmed H.A., Hagar M., Alhaddad O.A. (2019). Phase behavior and DFT calculations of laterally methyl supramolecular hydrogen-bonding complexes. Crystals.

[20] Ahmed H., Hagar M., Saad G. (2019). Impact of the proportionation of dialkoxy chain length on the mesophase behaviour of Schiff base/ester liquid crystals; experimental and theoretical study. Liq. Cryst..

[21] Hagar M., Ahmed H., El-Sayed T., Alnoman R. (2019). Mesophase behavior and DFT conformational analysis of new symmetrical diester chalcone liquid crystals. J. Mol. Liq..

[22] Alnoman R., Al-Nazawi F.K., Ahmed H.A., Hagar M. (2019). Synthesis, optical, and geometrical approaches of new natural fatty acids’ esters/Schiff base liquid crystals. Molecules.

[23] Hagar M., Ahmed H., Alhaddad O. (2019). New azobenzene-based natural fatty acid liquid crystals with low melting point: Synthesis, DFT calculations and binary mixtures. Liq. Cryst..

[24] Ahmed H., Hagar M., Alhaddad O. (2019). Mesomorphic and geometrical orientation study of the relative position of fluorine atom in some thermotropic liquid crystal systems. Liq. Cryst..

[25] Zaki A.A., Ahmed H., Hagar M. (2018). Impact of fluorine orientation on the optical properties of difluorophenylazophenyl benzoates liquid crystal. Mater. Chem. Phys..

[26] Dave J.S., Bhatt H.S. (2012). Synthesis of liquid crystals with lateral methyl group and study of their mesomorphic properties. Mol. Cryst. Liq. Cryst..

[27] Thaker B.T., Kanojiya J.B., Tandel R.S. (2010). Effects of different terminal substituents on the mesomorphic behavior of some azo-schiff base and azo-esterbased liquid crystals. Mol. Cryst. Liq Cryst..

[28] Alamro F.S., Tolan D.A., El-Nahas A.M., Ahmed H.A., El-Atawy M.A., Al-Kadhi N.S., Aziz S.G., Shibl M.F. (2022). Wide Nematogenic Azomethine/Ester Liquid Crystals Based on New Biphenyl Derivatives: Mesomorphic and Computational Studies. Molecules.

[29] Ahmed H.A., Aboelnaga A. (2022). Synthesis and mesomorphic study of new phenylthiophene liquid crystals. Liq. Cryst..

[30] Al-Kadhi N.S., Alamro F.S., Popoola S.A., Gomha S.M., Bedowr N.S., Al-Juhani S.S., Ahmed H.A. (2022). Novel Imidazole Liquid Crystals; Experimental and Computational Approaches. Molecules.

[31] Alamro F.S., Al-Kadhi N.S., Gomha S.M., Popoola S.A., Khushaim M.S., Alhaddad O.A., Ahmed H.A. (2022). Experimental and Theoretical Investigations of Three-Ring Ester/Azomethine Materials. Materials.

[32] El-Atawy M., Alhaddad O.A., Ahmed H.A. (2021). Experimental and geometrical structure characterizations of new synthesized laterally fluorinated nematogenic system. Liq. Cryst..

[33] Dave J.S., Menon M. (2000). Azomesogens with a heterocyclic moiety. Bull. Mater. Sci..

[34] Prajapati K., Pandya H. (2002). Azomesogens with 1,2,4–trisubstituted benzene moiety. Bull. Mater. Sci..

[35] Prajapati K., Pandya H. (2005). Azomesogens with methoxyethyl tail: Synthesis and characterization. J. Chem. Sci..

[36] Uhood J.A., Gassim T., Radhy H. (2010). Synthesis and characterization of a substituents on their liquid crystalline behavior. Molecules.

[37] Ahmed H.A., El-Atawy M.A., Alamro F.S., Al-Kadhi N.S., Alhaddad O.A., Omar A.Z. (2022). Mesomorphic, Computational Investigations and Dyeing Applications of Laterally Substituted Dyes. Molecules.

[38] Yeap G.Y., Ha S.T., Lim P.L., Boey P.L., Ito M.M., Sanehisa S., Youhei Y. (2006). Synthesis, physical and mesomorphic properties of Schiff base esters containing ortho-, meta-, and para-substituents in benzylidene-4′-alkanoyloxyanilines. Liq. Cryst..

[39] Ha S.-T., Ong L.-K., Wong J.P.-W., Yeap G.Y., Lin H.-C., Ong S.-T., Koh T.-M. (2009). Mesogenic Schiff’s base ether with dimethylamino end group. Phase Transit..

[40] Bhatt H.S., Patel P.D., Dave J.S. (2013). Study of mixed mesomorphism in binary systems of azo-ester mesogens with structurally dissimilar nonmesogenic as well as mesogenic ester homologues. Mol. Cryst. Liq. Cryst..

[41] Naoum M.M., Fahmi A.A., Abaza A.H., Saad G.R. (2014). Effect of exchange of terminal substituents on the mesophase behaviour of some azo/ester compounds. Liq. Cryst..

[42] Ahmed H.A. (2015). Mesophase stability in binary mixtures of monotropic nematogens. Liq. Cryst..

[43] Alshabanah L.A., Al-Mutabagani L.A., Gomha S.M., Ahmed H.A., Popoola S.A., Shaban M. (2021). Novel sulphonic acid liquid crystal derivatives: Experimental, computational and optoelectrical characterizations. RSC Advances.

[44] Kumar A., Varshney D., Prakash J. (2020). Role of ionic contribution in dielectric behaviour of a nematic liquid crystal with variable cell thickness. J. Mol. Liq..

[45] Alamro F.S., Ahmed H.A., Bedowr N.S., Naoum M.M., Mostafa A.M., Al-Kadhi N.S. (2022). New Liquid Crystals Based on Terminal Fatty Chains and Polymorphic Phase Formation from Their Mixtures. Crystals.

[46] Varshney D., Kumar A., Prakash J., Meena R., Asokan K. (2020). Gamma irradiation induced dielectric modulation and dynamic memory in nematic liquid crystal materials. J. Mol. Liq..

[47] Alamro F.S., Ahmed H.A., Bedowr N.S., Khushaim M.S., El-Atawy M.A. (2022). New advanced liquid crystalline materials bearing bis-azomethine as central spacer. Polymers.

[48] Yeap G.-Y., Osman F., Imrie C.T. (2015). Non-symmetric dimers: Effects of varying the mesogenic linking unit and terminal substituent. Liq. Cryst..

[49] Yeap G.-Y., Hng T.-C., Yeap S.-Y., Gorecka E., Ito M.M., Ueno K., Okamoto M., Mahmood W.A.K., Imrie C.T. (2009). Why do non-symmetric dimers intercalate? The synthesis and characterisation of the α-(4-benzylidene-substituted-aniline-4′-oxy)-ω-(2-methylbutyl-4′-(4″-phenyl) benzoateoxy) alkanes. Liq. Cryst..

[50] Imrie C.T., Henderson P.A. (2002). Liquid crystal dimers and oligomers. Curr. Opin. Colloid Interface Sci..

[51] Imrie C.T., Henderson P.A. (2007). Liquid crystal dimers and higher oligomers: Between monomers and polymers. Chem. Soc. Rev..

[52] Park J.W., Bak C.S., Labes M.M. (1975). Effects of molecular complexing on the properties of binary nematic liquid crystal mixtures. J. Am. Chem. Soc..

[53] Al-Mutabagani L.A., Alshabanah L.A., Gomha S.M., Ahmed H.A. (2021). Synthesis, thermal and optical characterizations of new lateral organic systems. Crystals.

[54] Araya K., Matsunaga Y. (1980). Liquid Crystal Formation in Binary Systems. I. An Interpretation of the Phase Diagrams of the Azoxydianisole–Schiff Base Systems. Bull. Chem. Soc. Jpn..

[55] Griffin A.C., Fisher R.F., Havens S.J. (1978). Effect of molecular structure on mesomorphism. 7. Enhancement of smectic-isotropic transition temperatures in binary mixtures of a new liquid crystal series: The 4-nitrophenyl 4’-n-alkoxybenzoates. J. Am. Chem. Soc..

[56] Radwan S.S., Sorkhoh N.A. (1993). Lipids of n-alkane-utilizing microorganisms and their application potential. Advances in Applied Microbiology.

[57] Khan M.T., Almohammedi A., Kazim S., Ahmad S. (2020). Electrical methods to elucidate charge transport in hybrid perovskites thin films and devices. Chem. Rec..

[58] Khan M.T. (2023). Unraveling the impact of graphene nanostructures passivation on the electrical properties of the perovskite solar cell. Mater. Sci. Semicond. Process..

[59] Jacquemin D., Laurent A.D., Perpète E.A., André J.-M. (2009). An Ab Initio Simulation of the UV/Visible Spectra of N-Benzylideneaniline Dyes. Int. J. Quantum Chem..

[60] Ebara N. (1960). Benzylideneaniline. I. Structure and Ultraviolet Absorption Spectrum of Benzylideneaniline. Bull. Chem. Soc. Jpn..

[61] Almohammedi A., Khan M.T., Benghanem M., Aboud S.W., Shkir M., AlFaify S. (2020). Elucidating the impact of PbI_2_ on photophysical and electrical properties of poly(3-hexythiophene). Mater. Sci. Semicond. Process..

[62] Khan M.T., Shkir M., Alhouri B., Almohammedi A., Ismail Y.A. (2022). Modulation of optical, photophysical and electrical properties of poly(3-hexylthiophene) via Gd:CdS nanoparticles. Optik.

[63] Khan M.T., Bajpai M., Kaur A., Dhawan S., Chand S. (2010). Electrical, optical and hole transport mechanism in thin films of poly (3-octylthiophene-co-3-hexylthiophene): Synthesis and characterization. Synth. Met..

[64] Deng X., Wen X., Zheng J., Young T., Lau C.F.J., Kim J., Green M., Huang S., Ho-Baillie A. (2018). Dynamic study of the light soaking effect on perovskite solar cells by in-situ photoluminescence microscopy. Nano Energy.

[65] Khan M.T., Almohammedi A., Shkir M., Aboud S.W. (2021). Effect of Ag_2_S nanoparticles on optical, photophysical and electrical properties of P3HT thin films. Luminescence.

