# Supplementary material for: Design of Liquid Crystal Materials Based on Palmitate, Oleate, and Linoleate Derivatives for Optoelectronic Applications
Source: Molecules. 2023 Feb 11;28(4):1744. doi: 10.3390/molecules28041744 (PMC9964637; doi:10.3390/molecules28041744)
Supplement: Supplementary file 1 [file molecules-28-01744-s001.zip › molecules-2180488-supplementary.pdf]

Supplementary Materials

Designing of liquid crystal materials based on palmitate, oleate, and linoleate derivatives for the optoelectronic application

Salma A. Al-Zahrani[1], Mohd Taukeer Khan[2], Violeta Jevtovic[1], Najat Masood[1], Yassin Aweis Jeilani[1] and Hoda A. Ahmed [3,4,*]

1 Chemistry Department, Faculty of Science, University of Ha'il, P.O. Box 2440, Ha'il 81451, Saudi Arabia
2 Department of Physics, Faculty of Science, Islamic University of Madinah, Al-Madinah Al-Munawwarah 42351, Saudi Arabia
3 Department of Chemistry, Faculty of Science, Cairo University, Cairo 12613, Egypt
4 Chemistry Department, Faculty of Science, Taibah University, Yanbu 46423, Saudi Arabia.

* Correspondence: H A. A; ahoda@sci.cu.edu.eg ; hmahmoud@taibahu.edu.sa

1. *Materials*

4-Chlorobenzaldyde, 4-methoxybenzaldyde, oleic acid, linoleic acid and 4-hydroxyaniline were obtained from Sigma Aldrich (Germany). N,N'-dicyclohexylcarbodiimide (DCC), 4-dimethylaminopyridine (DMAP), dichloromethane, ethanol and methanol were purchased from Aldrich (Wisconsin, USA). All chemicals were used without further purification.

2. *Synthesis of 4-substituted benzylideneamino)phenol:*

Equimolar amounts of 4-substitutedbenzaldehyde and 4-aminophenol (4.1 mmol each) in ethanol (10 ml) were refluxed for two hours. The reaction mixture was allowed to cool, and the separated product filtered. The obtained solid was recrystallized from ethanol.

3. *Synthesis of fatty acid derivatives* , **Ia-c** and **IIa-c**

Equimolar equivalents of (4-substitutedbenzylideneamino) phenol (4.1 mmol) and 4-fatty acids (4.1 mmol each) were dissolved in 25 ml dry methylene chloride. *N*, *N′*–dicyclohexylcarbodiimide (DCC, 0.02 mole) and a few crystals of 4–dimethylaminopyridine (DMAP) as a catalyst were added. The mixture was left to

stand for 72 hours at room temperature with continuous stirring. The solid residue obtained was filtered off and recrystallized twice from ethanol to give TLC pure products, indicated by TLC as one clear spot in their TLC chromatogram and sharp melting and clearing peaks in their differential scanning calorimetry (DSC) thermograms. The method previously reported in our work [45].

4. *Characterization*

TLC was performed with TLC sheets coated with silica gel (E. Merck); spots were detected by UV irradiation.

Varian EM 350L 500 MHz spectrometer (Oxford, UK) was used for recording ^{1}HNMR spectra using tetramethyl silane as internal standard in $CDCl_3$; the chemical shift values recorded as δ (in ppm units). Thermo Scientific Flash 2000 CHS/O Elemental Analyzer, Milan, Italy was used for Elemental analyses.

TA Instruments Co. (Q20 Differential Scanning Calorimeter, DSC; USA) was used for recording phase transitions. DSC calibration was carried out using lead and indium to calibrate the melting temperatures and enthalpies. Samples of 2–3 mg were used in aluminum pans for DSC investigation. The heating rate was 10°C/min in nitrogen gas as an inert atmosphere (30 ml/min). All transitions temperatures were measured from the second heating scan from the room temperature to 300 ºC on heating then cooled to 0 ºC.

Transition temperatures for the prepared compounds were checked and phases identified by Polarized optical microscope (POM, Wild, Germany) attached with Mettler FP82HT hot stage.
